# Six-Month Response to Delamanid Treatment in MDR TB Patients 

**DOI:** 10.3201/eid2310.170468

**Published:** 2017-10

**Authors:** Cathy Hewison, Gabriella Ferlazzo, Zaza Avaliani, Armen Hayrapetyan, Sylvie Jonckheere, Zarema Khaidarkhanova, Erika Mohr, Animesh Sinha, Alena Skrahina, Debrah Vambe, Irina Vasilyeva, Nathalie Lachenal, Francis Varaine

**Affiliations:** Médecins Sans Frontières, Paris, France (C. Hewison, F. Varaine);; Médecins Sans Frontières, Cape Town, South Africa (G. Ferlazzo, E. Mohr);; National Center for Tuberculosis and Lung Diseases, Tbilisi, Georgia (Z. Avaliani);; National Tuberculosis Control Centre Ministry of Health, Yerevan, Armenia (A. Hayrapetyan);; Médecins Sans Frontières, Johannesburg, South Africa (S. Jonckheere);; Republican Tuberculosis Dispensary, Grozny, Chechnya, Russian Federation (Z. Khaidarkhanova);; Médecins Sans Frontières, Minsk, Belarus (A. Sinha);; Republican Research and Practical Centre for Pulmonology and Tuberculosis, Minsk (A. Skrahina);; Swaziland National Tuberculosis Control Program, Mbabane, Swaziland (D. Vambe);; Research Institute of Phthisiopulmonology of I.M. Sechenov First MSMU, Moscow, Russian Federation (I. Vasilyeva);; Médecins Sans Frontières, Geneva, Switzerland (N. Lachenal)

**Keywords:** Tuberculosis, multidrug resistance, delamanid, drug therapy, combination, antitubercular agents, bacteria, antimicrobial resistance, MDR TB, Armenia, Belarus, Georgia, India, Russia, South Africa, Swaziland

## Abstract

Delamanid, recently available for the treatment of multidrug-resistant tuberculosis (MDR TB), has had limited use outside clinical trials. We present the early treatment results for 53 patients from 7 countries who received a delamanid-containing treatment for MDR TB. Results show good tolerability and treatment response at 6 months.

Outcomes of conventional 18–24-month regimens for multidrug-resistant tuberculosis (MDR TB) (*1*,*2*) and extensively drug-resistant tuberculosis (XDR TB) (*3*,*4*) are notoriously poor. Two recently marketed drugs, delamanid (*5*–*7*) and bedaquiline (*8*), represent hope for better outcomes. Médecins Sans Frontières (MSF) supported national TB programs to introduce delamanid according to World Health Organization recommendations (*9*) for patients lacking 4 effective second-line drugs in the regimen or at high risk for poor treatment outcomes. Delamanid was preferred over bedaquiline to treat TB in patients with hepatitis C (because of less potential hepatic toxicity with delamanid), patients who are taking antiretroviral drugs (because delamanid produces fewer interactions), or patients previously exposed to bedaquiline (and who had previous treatment failure) or clofazimine (because of potential cross resistance with bedaquiline). We present interim treatment response and safety data for patients treated with delamanid within MSF-supported programs.

This retrospective study comprises all patients started on MDR TB regimens containing delamanid in MSF-supported sites before March 1, 2016. Routine programmatic data were collected on site. Information on serious adverse events (SAEs) was retrieved from a central pharmacovigilance database. The study was approved by the relevant health ministries and meets the criteria of the MSF Ethics Review Board for exemption from ethics review.

We defined culture conversion as 2 consecutive negative culture results 1 month apart for culture-positive patients at start of delamanid treatment. We defined patients as having a favorable interim treatment response at 6 months if they completed 24 weeks of delamanid and culture converted or remained culture negative; we classified patients who did not meet these criteria as having an unfavorable interim treatment response. We used unadjusted bivariate odds ratios with 95% CIs to express the magnitude and precision of associations between outcomes and risk factors (the small number of records precluded a multivariable analysis). We defined SAEs as deaths irrespective of cause, hospitalizations, events leading to disability or congenital malformation, and events considered life threatening or otherwise medically noteworthy.

During February 6, 2015–February 29, 2016, a total of 53 patients from 7 countries (Technical Appendix Table 1) started a delamanid-containing regimen (Table). Of these, 46 (86.8%) received delamanid through a compassionate-use program. Most patients had been treated previously with second-line drugs (48/53, 90.6%), experienced MDR TB treatment failures (32/53, 60.4%), exhibited resistance to second-line TB drugs (41/51, 80.4%), or had extensive pulmonary disease (40/45, 88.9%). Almost all patients (52/53, 98.1%) received delamanid for an indication of <4 effective drugs in the regimen.

A total of 31 SAEs were reported in 14 patients (26.4%); most common were hepatotoxicity (5), electrolyte imbalance (5), and QT prolongation (3). The most frequent contributing factors reported were TB disease (6), hepatitis C infection (6), and non–anti-TB drugs, including antiretroviral drugs (ARVs) (8). A possible relation to any TB drug was reported in 80.6% (25/31) of events, including a possible relation to delamanid in 58.6% (18/31). Causes of the 7 reported deaths were advanced TB (2), encephalitis in an untreated HIV patient (1), traumatic pneumothorax (1), sepsis in an HIV patient (1), respiratory failure related to end-stage hepatitis (1), and sudden death of unknown cause (1); a possible relationship to anti-TB drugs was initially reported in the last 2 cases. In 1 patient with hepatitis C and liver cirrhosis, all drugs were permanently discontinued due to hepatotoxicity. No other permanent discontinuation of delamanid was reported (Technical Appendix Table 2). 

Of the patients who were culture positive at delamanid start, 67.6% (25/37) culture converted by 6 months. At 6 months, 73.6% (39/53) of patients had a favorable response, 13.2% (7/53) had died, 7.5% (4/53) remained culture positive, 3.8% (2/53) were lost to follow-up, and 1.9% (1/53) were declared to have a failure in treatment as a result of an SAE. Factors associated with unfavorable response in a univariate analysis were age >35 years (odds ratio [OR] 5.62, 95% CI 1.47–21.57; p = 0.012); hepatitis C infection (OR 7.78, 95% CI 1.45–41.78; p = 0.017); smear positivity at delamanid start (OR 5.21, 95% CI 1.35–20.06; p = 0.016); and serum albumin <34 g/L (OR 7.14, 95% CI 1.6–33.3; p = 0.010) (Technical Appendix Table 3).

These preliminary results indicate good tolerability and interim treatment response to delamanid at 6 months in a narrow and difficult-to-treat cohort of patients for whom delamanid was preferred to bedaquiline, most of whom had previously failed MDR TB treatment and had extensive disease. Delamanid was used in preference to bedaquiline in this group of patients, despite the programmatic availability of bedaquiline, which may explain the frequency of adverse events in relation to hepatitis C and HIV co-infection, comorbidities that influence this choice, further supporting the need for essential monitoring and treatment of hepatitis C and HIV in MDR TB patients. Limitations of this study include its small numbers and retrospective nature, and data on delamanid treatment outcomes and safety in programmatic conditions with larger indications deserve further studies.

**Table:** Demographic, clinical, and bacteriological characteristics at baseline of 53 patients starting a delamanid-containing MDR TB treatment regimen*

Variable	No. (%) patients or median (IQR)
Sex	
M	36 (67.9)
F	17 (32.1)
Age at delamanid start, y	29.5 (20.0–43.0)
14–17	11 (20.8)
HIV co-infected, n = 48	8 (16.7)
HCV co-infected, n = 42	8 (19.0)
Malnutrition,† n = 51	21 (41.2)
Serum albumin at delamanid start, g/L, n = 46	37.6 (32.0–37.6)
WHO case definition	
New case	4 (7.5)
Relapse	5 (9.4)
Treatment after being lost to follow-up	5 (9.4)
Treatment after failure	32 (60.4)
Other	7 (13.5)
Previously treated	49 (92.4)
With first-line drugs only	1 (2.1)
With second-line drugs	48 (97.9)
MDR TB confirmed	51 (96.2)
Drug resistance subgroups among confirmed MDR TB	
MDR TB only‡	10 (19.6)
Pre–XDR TB FQ	6 (11.8)
Pre–XDR TB Inj	8 (15.7)
XDR TB	27 (52.9)
Radiograph features	
Bilateral, n = 45	35 (77.8)
Cavities, n = 43	26 (60.5)
Bilateral or cavity, n = 45	40 (88.9)
Culture positive at delamanid start	37 (69.8)
*HCV, hepatitis C virus serology; HIV, human immunodeficiency virus; MDR TB, multidrug-resistant tuberculosis; pre–XDR TB FQ, MDR TB with fluoroquinolone resistance; pre–XDR TB Inj, MDR TB with resistance to injectable drugs; WHO, World Health Organization; XDR TB, extensively drug-resistant tuberculosis. †Malnutrition: either BMI <18.5 kg2/cm, mid-upper arm circumference <16cm, or weight <50 kg in 3 patients from South Africa without height measurement. ‡Without resistance to fluoroquinolone or injectable drugs.	

Technical Appendix The pharmacovigilance system of reporting serious adverse drug effects and additional patient information for study of delamanid-containing multidrug-resistant tuberculosis treatment regimen.
