# Supplementary material for: Six-Month Response to Delamanid Treatment in MDR TB Patients
Source: Emerg Infect Dis. 2017 Oct;23(10):1746–8. doi: 10.3201/eid2310.170468 (PMC5621551; doi:10.3201/eid2310.170468)
Supplement: Technical Appendix — The pharmacovigilance system of reporting serious adverse drug effects and additional patient information for study of delamanid-containing multidrug-resistant tuberculosis treatment regimen. [file 17-0468-Techapp-s1.pdf]

Article DOI: https://doi.org/10.3201/eid2310.170468

Six-Month Response in MDR TB Patients Treated with Delamanid

Technical Appendix

Pharmacovigilance System of Reporting Serious Adverse Events

All sites mentioned in the report performed active pharmacovigilance (PV) reporting on serious adverse events (SAEs). The site clinicians were trained on systematic collection and reporting of SAEs by the central MSF PV unit (Geneva) either through on site or virtual (web conference) training before the introduction of delamanid-containing regimens. Clinicians were specifically trained on detection of SAEs and on the tools allowing for the recording and reporting of such safety information, including MSF PV guidelines, report forms, and the severity grading scale for the project (Division of Microbiology and Infectious Diseases adult toxicity table, completed by Common Terminology Criteria for Adverse Events, Division of AIDS , and other scales).

SAEs were notifiable to the MSF PV unit within 24 hours of knowledge at the site. The MSF OV unit entered detailed information, including patient demographics and relevant medical background, SAE description, TB regimen information and concomitant drugs, relevant laboratory test results, treating physician’s causality assessment, and reporter details, into a central PV database based on the forms transmitted by the sites. The PV unit followed up cases until the SAE outcome was known and organized the medical review by a central team physician for each individual case. Both local and central assessments co-exist in the database; cases judged relevant were additionally reviewed for opinion and signal detection purpose by an expert medical committee at central level (the Medical Review Board).

Copies of SAE report forms were shared with the national authorities by the field clinicians as applicable per national laws. The central PV unit additionally shared SAE information with drug manufacturers, enabling further dissemination to the European Medicine Agency or the US Food and Drug Administration (among other health authorities).

Clinical patient data on treatment including efficacy and safety information were recorded at local site level in local electronic medical record databases; SAE data were reconciled between the local databases and the central PV database. Safety data for this study were taken from the central PV unit database after reconciliation.

Details about patients who developed hepatotoxicity, SAEs of QTcF increase, and SAES with fatal outcomes initially reported as possibly related to TB drugs (see Technical Appendix Table 2) follow.

Details of Hepatoxicity SAEs

Two episodes of hepatotoxicity occurred in 1 patient coinfected with active hepatitis C with cirrhosis (genotype 3 HCV, fibroscan, 13.1 Kpa), which led to permanent discontinuation of all TB drugs, including delamanid and bedaquiline, and a treatment outcome of failure (first episode on bedaquiline: ALT 243 U/L, AST 348 U/L, second episode on delamanid: ALT 304 U/L, AST 241 U/L). The patient was later treated for hepatitis C with direct-acting antiviral drugs (sofosbuvir, daclatasvir, and ribavirin).

In 1 patient co-infected with HIV and on ARVs who had previously had hepatitis C infection but was PCR negative for hepatitis C, all drugs were stopped temporarily. Delamanid was continued with other TB drugs when the hepatoxicity, which was thought to be related to ARVs and prior hepatitis C (ALT 154 U/L, AST 1075 U/L), resolved. The outcome of the hepatoxicity is unknown.

In 1 patient with active hepatitis C (genotype 2, fibroscan metavir score was F0–F1), delamanid was continued after temporarily stopping all drugs. Drug challenge found hepatotoxicity related to other TB drugs (AST 300 IU/L, ALT 461 IU/L). Liver enzymes increased during February 9, 2015–July 4, 2015 until full resolution; however, liver enzymes were not normal at treatment start and recurrent increases and fluctuations occurred during this time as a result of drug challenges.

In 1 patient without any coinfection with HIV or hepatitis C, all drugs were stopped. Drug challenge found hepatotoxicity related to other drugs, and treatment was restarted with delamanid (ALT 183.6 U/L, AST 157.2 U/L). Liver enzymes increased during September 14, 2015–April 14, 2016 for full resolution; however, there were recurrent liver enzyme increases and fluctuations during this time due to drug challenges.

No patients received direct-acting antiviral therapy for active hepatitis C treatment during MDR TB treatment.

Details of QTcF Increase SAEs

Three patients experienced serious adverse events of QTcF increase.

Patient 1: QtcF increase of 592 msec, associated with severe vomiting, severe hypokalemia, moderate hypocalcemia, hypomagnesemia, and hypoalbuminemia; all TB drugs (delamanid-Cm-Eto-Cfz-cs-Lzd-Z-E-Imp-Amox/Clav) and ondansetron were stopped. Adjusted TB treatment including delamanid was restarted after correction of electrolyte imbalances.

Patient 2: QtcF increase of 557 msec, associated with hypoalbuminemia, hypomagnesemia, hypokalemia, nephrotic syndrome, long-standing anemia, and extensive XDR-TB. TB treatment was stopped (delamanid-Cm-Lzd-Cfz-Imp-Amox/Clav-Eto-Cs-Rfb) and reintroduced after correction of electrolytes. The patient later died as a result of complications of traumatic pnuemothorax.

Patient 3: QtcF increase of 510 msec while on TB treatment of bedaquiline-Lzd-PAS-Cfz-Lfx-Trd before starting delamanid associated with vomiting, sepsis, and renal failure. The patient was on ARV treatment of tenofovir, emtricitabine, and efavirenz and later died as a result of complications of sepsis.

Details of SAES with Fatal Outcomes Initially Reported as Possibly Related to TB Drug

One patient experienced cardiogenic shock and respiratory failure: a 47-year-old man who died 36 days after starting delamanid-containing XDR-TB treatment (delamanid- Cfz-Lzd-Lfx-Cs-Cm), in severe condition related to TB and advanced liver disease due to hepatitis C (with cirrhosis, ascites, and encephalopathy). The patient had renal failure, anemia, and prior QT prolongation requiring Cfz discontinuation (17 days before death), and developed respiratory insufficiency and hypotension; the patient died as a result of cardiogenic shock and respiratory insufficiency. This case was initially conservatively considered by the treating physician as possibly related to all drugs. After the data lock point for this analysis and in the light of all the details on the patient’s medical history, the treating physician and the central PV review agreed that the events were unlikely to be related to the TB drugs and rather were secondary to the patient’s comorbidities, namely hepatitis C cirrhosis and TB.

The second patient was a 21-year-old man who died 21 days after starting adapted delamanid-containing XDR-TB treatment (delamanid-Imp-Amx/Clv added to Cm-Mfx-PAS-Lzd administered for 75 days at time of death). Central venous catheter was placed 3 days before the death. About 1 hour after drug infusion on the day of the death, the patient was at home and developed dyspnea, restlessness, anxiety, and sweating, as reported by his parents. He was brought to a nearby hospital, where he was declared stable and the decision was made to transfer him to another facility (presumably for infection control reasons). The patient went into cardiorespiratory arrest en route to the hospital and was declared dead upon arrival. Retrospectively, the case was reviewed and hypothetical causes of death included pulmonary embolism, pneumothorax/tension pneumothorax, or cardiac arrhythmia. None of these hypotheses could be fully confirmed with the available information. Conservatively, the death was considered of unknown cause and recorded as potentially related to any drug in the absence of sufficient information to fully assess the case.

Technical Appendix Table 1. Country of residence of patients with MDR TB who received delamanid

Country	N (%)
Armenia	8 (15.1)
Belarus	6 (11.3)
Georgia	12 (22.6)
India	11 (20.8)
Russia	8 (15.1)
South Africa	6 (11.3)
Swaziland	2 (3.8)

Technical Appendix Table 2. Serious adverse event terms; frequency; relation to TB drugs, delamanid and non-TB drugs; other causal factors; comorbidities; and outcomes*

SAE term	N	Associated factors	Possibly related to TB drugs	Possibly related to delamanid	Possibly related to non-TB drugs	HIV	Advanced TB	HepC	Outcomes
Electrolyte imbalance: hypokalemia (2), hypomagnesemia (2), hypocalcemia (1)	5	3 associated with ondansetron, omeprazole	5	5	3	0	NA	NA	All resolved/ recovered
Hepatotoxicity (raised transaminases, drug-induced liver injury)	5	4 with HCV, 1 with ARVs	5	3	1	0	NA	4	4 recovered/ resolved, 1 unknown†
End-stage TB (tuberculosis, pulmonary hemorrhage, asphyxia)	3	3 Advanced TB	0	0	0	0	3	NA	3 fatal
QTcF prolongation	3	2 associated with electrolyte imbalance, 1	3	3	1	0	NA	NA	3 resolved/ recovered†

SAE term	N	Associated factors	Possibly related to TB drugs	Possibly related to delamanid	Possibly related to non-TB drugs	HIV	Advanced TB	HepC	Outcomes
		with non-TB drugs: ondansetron, omeprazole							
Hypoalbuminemia	2	1 non-TB drugs: ondansetron, omeprazole	2	2	1	0	NA	NA	2 resolved/ recovered
Encephalitis	1	Untreated HIV	0	0	0	1	NA	NA	Fatal
Cardiogenic shock and respiratory failure	2	2 advanced hepC and cirrhosis	2	2 (initially)	0	0	1	2	Fatal†
Psychotic disorder	1	Possible tuberculoma or vasculitis	1	0	0	0	1	NA	Not recovered/ not resolved
Systematic inflammatory response	1	TB, other infection	1	0	0	0	NA	NA	Recovered/ resolved
Nephrotic syndrome	1	NA	1	1	0	0	NA	NA	Recovered/ resolved
Renal failure	1	TDF and vomiting/ dehydration	1	0	1	0	NA	NA	Recovering/ resolving
Traumatic pneumothorax	1	Insertion of portacath	0	0	0	0	NA	NA	Fatal
Sepsis	1	Prolonged hospital stay in HIV patient	0	0	0	1	NA	NA	Fatal
Vomiting	1	ARVs	1	0	1	0	NA	NA	Recovered/ resolved
Weight loss	1	Failure of DRTB treatment	1	1	0	0	1	NA	Unknown
Death	1	NA	1	1	0	0	NA	NA	Fatal†
Device-related infection	1	NA	1	0	0	0	NA	NA	Recovered/ resolved
Total	**31**		**25**	**18**	**8**	**2**	**6**	**6**	
%			80.6%	58.1%					

*ARVs, antiretroviral drugs; DRTB, drug-resistant tuberculosis; HepC, hepatitis C; NA, not applicable; SAE, serious adverse effect; TDF, tenofovir disoproxil fumarate
†Details of hepatotoxicity SAEs, QtcF prolongation SAEs, and SAEs with fatal outcomes initially assessed as possibly being related to delamanid are described in the Technical Appendix text.

Technical Appendix Table 3. Factors associated with unfavorable response in a univariate analysis of patients with MDR TB*

Characteristics	Unfavorable outcome n (%)	OR	95% CI	p-value
Age				
<35	4/31 (12.9)	**1**		
≥35	10/22 (45.5)	**5.62**	**1.47–21.57**	**0.012**
Sex				
Male	11/36 (30.6)	1		
Female	3/17 (17.7)	0.49	0.12–2.04	0.326
HIV				
Negative	10/40 (25.0)	1		
Positive	4/8 (50.0)	3.0	0.63–14.27	0.167
Hepatitis C				
Negative	6/34 (17.7)	**1**		
Positive	5/8 (62.5)	**7.78**	**1.45–41.78**	**0.017**
Malnutrition†				
Good	7/30 (23.3)	1		
Poor	5/21 (23.8)	1.02	0.28–3.82	0.969
X-ray changes				
Unilateral	0/10 (0.0)			
Bilateral	9/35 (25.7)	NA	NA	NA
Cavity on X-ray				
No	1/17 (5.9)	1		
Yes	7/26 (26.9)	5.90	0.65–53.11	0.114
Smear at delamanid start				
Negative	4/29 (13.8)	**1**		
Positive	10/22 (45.5)	**5.21**	**1.35–20.06**	**0.016**
Culture at delamanid start				
Negative	2/16 (12.5)	1		
Positive	12/37 (32.4)	3.36	0.66–17.21	0.146
DST profile				
MDR, pre-XDR (Inj, FQ)	6/24 (25.0)	1		
XDR	8/27 (29.6)	4.18	0.48–36.53	0.196
Albumin at delamanid start				
<34	7/14 (50.0)	**1**		
≥34	4/32 (12.5)	**0.14**	**0.03–0.63**	**0.010**
Cfz in regimen				
No	5/12 (41.7)	1		
Yes	7/36 (19.4)	0.34	0.08–1.39	0.133
Mfx in regimen				
No	9/31 (29.0)	1		
Yes	1/15 (6.7)	0.17	0.02–1.53	0.115

*Bold text indicates figures with $p < 0.05$. Cfz, clofazimine; DST, drug susceptibility testing; HIV: human immunodeficiency virus; MDR, MDR TB without resistance to fluoroquinolone or injectable drugs; MDR TB, multidrug-resistant tuberculosis; Mfx: moxifloxacin; pre-XDR-TB (FQ): MDR TB with fluoroquinolone resistance; pre-XDR-TB (Inj): MDR TB with resistance to injectable drugs; XDR-TB, extremely drug-resistant tuberculosis
†Malnutrition: either BMI <18.5 kg^2/cm, mid-upper arm circumference <16 cm, or weight <50 kg in 3 patients from South Africa without height measurement

*
